# Extracellular SOD and VEGF are increased in vitreous bodies from proliferative diabetic retinopathy patients

**Published:** 2009-12-10

**Authors:** Hiroshi Izuta, Yuichi Chikaraishi, Tetsuo Adachi, Masamitsu Shimazawa, Tetsuya Sugiyama, Tsunehiko Ikeda, Hideaki Hara

**Affiliations:** 1 Department of Biofunctional Evaluation, Molecular Pharmacology, Gifu Pharmaceutical University, Mitahora-higashi, Japan; 2 Department of Biomedical Pharmaceutics, Clinical Pharmaceutics, Gifu Pharmaceutical University, Mitahora-higashi, Japan; 3 Department of Ophthalmology, Osaka Medical College, Takatsuki, Japan

## Abstract

**Purpose:**

To evaluate the relationship between vascular endothelial growth factor (VEGF) and extracellular superoxide dismutase (EC-SOD) in vitreous body and serum in patients with proliferative diabetic retinopathy (PDR), and investigate the role of EC-SOD in PDR by evaluating its angiostatic effect, using an in vitro angiogenesis model. To investigate the role of EC-SOD in PDR by evaluating its angiostatic effect, using an in vitro angiogenesis model.

**Methods:**

EC-SOD and VEGF concentrations in vitreous and serum samples from PDR and macular hole (MH) were measured by ELISA. The effects of EC-SOD on VEGF-induced proliferation, migration, and tube formation were evaluated using human umbilical vein endothelial cells (HUVECs). Moreover, the effects of EC-SOD on VEGF-induced proliferation and migration were evaluated in HUVECs and primary normal human retinal microvascular endothelial cells.

**Results:**

Intravitreal concentrations of EC-SOD were significantly higher (p<0.01) in PDR (58.0±23.8 ng/ml, mean±SD) than in MH (29.3±6.6 ng/ml). Intravitreal concentrations of VEGF were dramatically higher (p<0.01) in PDR (798.2±882.7 pg/ml) than in MH (17.7±15.5 pg/ml). The serum concentrations of EC-SOD and VEGF did not differ between the two patient groups. The vitreous concentrations of VEGF correlated with those of EC-SOD in all patients (rs=0.61, p<0.001). In HUVECs, EC-SOD at 100 ng/ml significantly suppressed VEGF-induced proliferation and tube formation, but not VEGF-induced migration.

**Conclusions:**

EC-SOD was increased together with VEGF in the vitreous body from PDR patients, suggesting that EC-SOD may play a pivotal role in the pathogenesis of angiogenesis.

## Introduction

Proliferative diabetic retinopathy (PDR) is characterized by extensive neovascularization and vessel intrusion into the vitreous body, with subsequent bleeding around the new vessels that leads to severe visual impairment. This process depends on the local production of vascular endothelial growth factor (VEGF) and other angiogenic factors. VEGF, a potent activator of angiogenesis, enhances collateral vessel formation and increases the permeability of the microvasculature [1,2]. VEGF expression is induced by high glucose levels and by hypoxia, and this growth factor plays important roles in both normal and abnormal angiogenesis [3,4]. Its levels markedly increase in the vitreous and aqueous fluids in the eyes of patients with PDR [5,6].

Oxidative stress is defined as a condition in which tissue damage results from an imbalance between an excessive generation of oxidant compounds and inadequate antioxidant defense mechanisms [7]. In diabetic eye disease, oxidative stress clearly plays a key role in the initial insult, and there is a strong relationship between oxidative stress and hyperglycemia [8]. Oxidative stress has been correlated with an increased production of VEGF under in vitro conditions, and is thought to be involved in the upregulation of VEGF expression that occurs during diabetes [9,10]. In addition, several studies involving animal and tissue culture models have indicated that oxidative stress is also a critical mediator in the transduction of the mitogenic effects of VEGF [11,12].

Antioxidant systems occur naturally within mammalian tissues, where they serve to protect against the harmful side effects of reactive oxygen species (ROS) by counteracting free radical reactions. Extracellular superoxide dismutase (EC-SOD), one of the SOD family enzymes, has the scavenging capacity of superoxide anion. The heparin-binding domain of EC-SOD anchors the protein to endothelial cell surfaces and to the extracellular matrix of blood vessels. Fattman and colleagues [13] have shown that EC-SOD activity is decreased in both anterior and posterior tibial arteries of diabetic patients. However, the relationship between the concentration of EC-SOD and its function in the vitreous body on PDR remains unclear.

The aim of our study was to investigate the alteration of EC-SOD concentration in the vitreous and serum from PDR patients. Macular hole (MH) patients served as controls. We also measured the levels of VEGF in vitreous body and serum samples obtained from patients with PDR. The function of EC-SOD as an angiogenesis antagonist was also investigated by examining the effects of EC-SOD in VEGF-induced proliferation, and wound healing assays in human umbilical vein endothelial cells (HUVECs) and human retinal microvascular endothelial cells (HRMVECs). Moreover, we investigated the effect of EC-SOD using in vitro tube formation in HUVECs.

## Methods

### Preparation of vitreous body and serum samples

This study was conducted according to the tenets of the Declaration of Helsinki and received approval from the institutional review committee of Osaka Medical College. Informed consent was obtained from all patients after an explanation of the purpose and procedures of the study.

A total of 12 PDR patients and 14 MH patients were recruited. The PDR patients included five men and seven women, who were 52.9±10.6 years old (mean±standard deviation, SD). The MH patients consisted of one man and 13 women, who were 63.5±10.6 years old. Undiluted vitreous samples were collected from 28 eyes of 26 individuals from 14 PDR patients and 14 MH patients, who were undergoing pars plana vitrectomy for the treatment of diabetic retinopathy (DR) and other retinal disorders at Osaka Medical College Hospital. Samples with repeat vitrectomy were excluded. Simultaneously, serum samples were collected from nine PDR patients and nine MH patients. The vitreous and serum samples were collected by same patients with PDR and MH. PDR patients with or without macular edema and traction membrane were included. MH patients without neovascular disease and vitreous hemorrhage were used as controls, because this disorder is caused by vitreomacular traction occurring before a posterior vitreous detachment and exhibits no signs of ischemia, proliferation or inflammation. Therefore, we believe that vitreous body from patients with MH is the most similar in constitution to normal eyes that can be obtained. Details of the patients with PDR and MH are shown in Table 1.

**Table 1:** Data for patients with proliferative diabetic retinopathy or macular hole.

**Characteristic**	**Macular hole** **(14 patients)**	**Proliferative diabetic retinopathy** **(12 patients)**
Age (years)	63.5±10.6	52.9±10.6
Number of women	13	7
Diabetes duration (years)	-	7.7±1.6
**Clinical findings**		
Macular edema	-	4
Proliferative membrane	-	1
Traction membrane	-	1
Alone	-	2
Vitreous hemorrhage	-	7
Traction membrane	-	6
Proliferative membrane	-	8
Tractional retinal detachment	-	5
Alone	-	3
**Pretreatments**		
Insulin	-	6
Hypoglycemic drug	-	6
Macular hole Stage 2	6	-
Macular hole Stage 3	8	-

The table indicates the baseline characteristics and the complications of proliferative diabetic retinopathy and macular hole patients. Complications of each patient are shown in “clinical findings,” and “pretreatments” indicates therapies until vitreous surgeries. In proliferative diabetic retinopathy, majority of complications were macular edema, vitreous hemorrhage, and proliferative membrane. 50% of the patients were pretreated with insulin, and the others were hypoglycemic drugs in proliferative diabetic patients. In macular hole patients, 6 patients were macular hole stage2, and 8 patients were macular hole stage3. “Age” and “Duration” data are mean±SD.

Before intraocular infusion of a balanced salt solution, the vitreous core was cut and aspirated via the pars plana, with a vitreous cutter. The vitreous body samples (0.6–0.8 ml) were spun for 10 min at 15,000× g in a refrigerated centrifuge at 4 °C to remove particles and then were stored in aliquots in polypropylene tubes at −80 °C until assay. Serum (2.0 ml) samples were also collected into sterile tubes simultaneously with vitreous surgery, and rapidly frozen at −80 °C.

### Cells and chemicals

HUVECs, fibroblast cells, VEGF-A, mouse anti-human CD31 antibody, goat anti-mouse IgG alkaline phosphatase-conjugated antibody, 5-bromo-4-chloro-3-indolyl phosphate/nitro blue tetrazolium (BCIP/NBT), and angiogenesis growth medium were all purchased from Kurabo (Osaka, Japan). HRMVECs, CS-C medium, a growth medium optimized for HRMVECs, and culture boost were purchased from DS Pharma Biomedical (Osaka, Japan). The cell culture kit-8 (CCK-8) was from Dojindo (Kumamoto, Japan).

### Cell cultures

HUVECs were cultured in HuMedia-EB2 (Kurabo) supplemented with 2% (v/v) fetal bovine serum (FBS), 50 µg/ml gentamicin, 50 ng/ml amphotericin B, and endothelial growth factors at 37 °C in a humidified atmosphere of 5% CO_2_ in air. The endothelial growth factors contained 10 ng/ml human epidermal growth factor (hEGF), 1 µg/ml hydrocortisone, 5 ng/ml human basic fibroblast growth factor (hFGF-B), and 10 µg/ml heparin. HRMVECs were cultured in CS-C medium supplemented with 10% (v/v) FBS, 50 µg/ml gentamicin, 50 ng/ml amphotericin B, and culture boost (growth factors) at 37 °C in a humidified atmosphere of 5% CO_2_ in air.

### Measurement of EC-SOD

To measure human EC-SOD, we used the ELISA method previously described [14]. An 80 ml portion of 50 mg/l monoclonal antibody dissolved in sodium carbonate buffer, 50 mM, pH 9.5, containing 0.02% sodium azide, was added to each well of the immunoplates and left to stand overnight at 4 °C. Each well was washed with sodium phosphate buffer, 10 mM, pH 7.4 containing 150 mM NaCl, 0.05% Tween-20 (washing buffer). The remaining protein-binding site were blocked with 300 ml of sodium phosphate buffer, 10 mM, pH 7.4 containing 150 mM NaCl, 1% BSA, 0.05% Tween-20 (blocking buffer). The plate was then left to stand at 4 °C until use. Sample or standard (70 ml) dilute d with the blocking buffer was added to the wells. The plate was incubated for 2 h at room temperature and washed three times with the washing buffer. Then 80 ml of alkaline phosphatase-labeled monoclonal antibody diluted with the blocking buffer were added to each well, and the plate was incubated for 2 h at room temperature, followed by washing three times with the washing buffer. Substrate solution (0.1 M diethanolamine hydrochloride, pH 9.8, containing 0.5 mM MgCl_2_, 0.02% sodium azide and 2.7 mM p-nitrophenyl phosphate) was then added to each well and the plate was incubated for 30 min at room temperature. The enzyme reaction was stopped by the addition of 50 ml of 5 M NaOH, and the absorbance at 415 nm was measured.

### Measurement of VEGF

The VEGF concentrations in vitreous body and serum were measured using a human VEGF ELISA kit (Pierce Biotechnology, Rockford, IL). In brief, 50 μl of sample diluent and 50 μl of either the standard control or a fivefold diluted vitreous body or serum sample were added to each well of the ELISA plate, incubated for 2 h at room temperature, and washed three times at room temperature (Endogen VEGF ELISA Kit). Then, 100 μl of anti-human VEGF biotinylated antibody reagent was added to each well, incubated for 1 h at room temperature, and washed three times at room temperature (Endogen VEGF ELISA Kit). Streptavidin- horseradish peroxidase (HRP) reagent was added to each well, incubated for 30 min at room temperature, and then washed three times at room temperature (Endogen VEGF ELISA Kit). Next, 100 μl TMB substrate solution was added to each well, and the plate was developed in darkness at room temperature for 30 min. Finally, 100 μl of stop solution was added, and concentrations were determined at 450 nm (correction 550 nm) using a microplate reader.

### Proliferation assay

HUVECs or HRMVECs were seeded at 2×10^3^ cells per well into a 96-well plate, then incubated for 24 h at 37 °C in a humidified atmosphere of 5% CO_2_ in air. HUVECs were rinsed twice with PBS (137 mM sodium chloride, 2.7 mM potassium chloride, 10.1 mM disodium hydrogen phosphate 12 hydrate, and 1.8 mM potassium dihydrogenphosphate), then exposed for 6 h to HuMedia-EB2 containing 2% FBS. HRMVECs were rinsed twice with PBS, then exposed for 6 h to CS-C medium containing 2% FBS. The HUVECs and HRMVECs were incubated with 10 ng/ml VEGF with or without 100 ng/ml EC-SOD. Cell viability was determined by CCK-8 to count living cells by combining 2-(2-methoxy-4-nitrophenyl)-3-(4-nitrophenyl) −5-(2,4-disulfophenyl)-2H-tetrazolium (WST-8) and 1-methoxy-phenazine methosulfate (1-methoxy-PMS).

### In vitro wound healing assay

An in vitro wound healing assay was performed to measure unidirectional migration of the endothelial cells. HUVECs or HRMVECs were seeded at 4×10^4^ cells per well into a 12-well plate and incubated for 48 h at 37 °C in a humidified atmosphere of 5% CO_2_ in air. Afterwards, HUVECs or HRMVECs were washed with PBS twice and incubated in HuMedia-EB2 or CS-C medium with 1% FBS. After 24 h incubation, the monolayers of the endothelial cells were scratch-wounded to a 1 mm depth in a straight line using a 10 to 200 µl micro-tip. The HUVECs/HRMVECs were then incubated with 10 ng/ml VEGF with or without 100 ng/ml EC-SOD for 24 h. Images were taken at the time of wounding and at 24-h intervals thereafter, using a phase-contrast microscope (Olympus, Tokyo, Japan). Migration was estimated by counting the cell numbers within the wounded region, and the migrating cells were counted in a masked fashion by a single observer (H.I.).

### In vitro tube formation assay

HUVECs and fibroblasts were cocultured in angiogenesis growth medium supplemented with 10 ng/ml VEGF with or without 100 ng/ml EC-SOD at 37 °C in a humidified atmosphere of 5% CO_2_ in air. In the control group, HUVECs were incubated with angiogenesis growth medium. This treatment was repeated every four days. After 11 days of incubation, cells were fixed with 70% ethanol, then stained with 1:4,000 mouse anti-human CD31 antibody for 1 h, and thereafter treated with 1:500 goat anti-mouse alkaline phosphatase-conjugated antibody for 1 h. BCIP/NBT solution was then applied until HUVECs were stained deep purple. Images were collected using a digital camera (CoolPix 4500; Nikon, Tokyo, Japan). Tube formation was estimated by measurements of joint and path using software for tube formation analysis (Kurabo). A joint is where two different tubes intersect each other. Path means the tubes that branch from a joint.

### Statistical analysis

Statistical analyses were performed with the aid of SPSS 15.0J for Windows software (SPSS Japan Inc., Tokyo, Japan). Data are presented as means±SD. Statistical comparisons of in vitro experiments were made using a one-way ANOVA (ANOVA) followed by a Tukey test. Statistical comparisons of clinical samples were made using a Kruskal–Wallis test and Spearman rank-correlation test. Spearman’s rho correlation coefficient was described as “rs.” A value of p<0.05 was considered to indicate statistical significance.

## Results

### EC-SOD levels in vitreous and serum samples from PDR and MH patients

**Figure 1:**
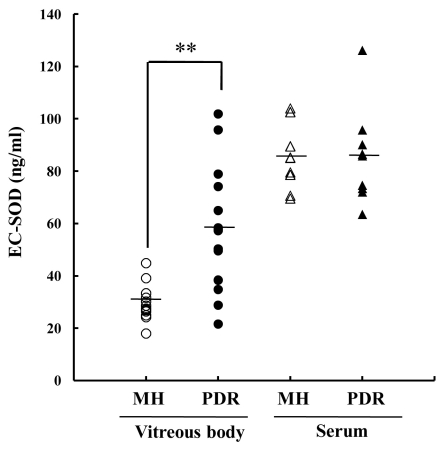
EC-SOD levels in vitreous body and serum samples from PDR and MH patients. In the vitreous body from PDR patients, there was abundant EC-SOD compared to vitreous bodies from MH patients. In contrast, the serum concentration of EC-SOD was not significantly different between the two groups. Double asterisk (**) denotes p<0.01 based on the Kruskal–Wallis test.

In each group, the levels of EC-SOD in vitreous and serum were measured using ELISA (Figure 1). Intravitreal levels of EC-SOD were significantly higher (p<0.01) in PDR (58.0±23.8 ng/ml, mean±SD) than in MH (29.3±6.6 ng/ml). In contrast, the serum levels of EC-SOD were not significantly different between the PDR group (85.3±18.4 ng/ml) and the MH group (85.0±12.3 ng/ml; p=0.96).

### VEGF levels in vitreous and serum samples from PDR and MH patients

**Figure 2:**
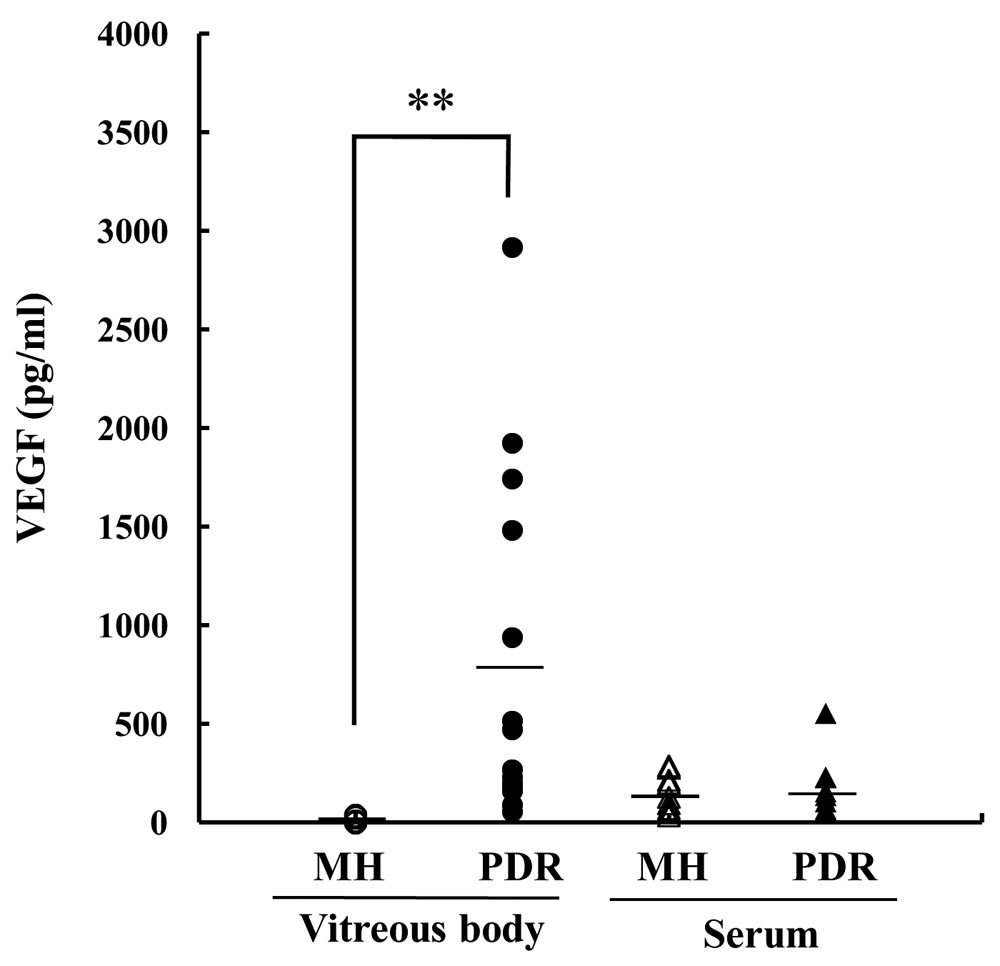
VEGF levels in vitreous body and serum samples from PDR and MH patients. The vitreous levels of VEGF were higher (p<0.01) in PDR (798.2±882.7 pg/ml; n=14) than in MH (17.7±15.5 pg/ml; n=14). On the other hand, the serum levels of VEGF were no significant differences (p=0.83) between PDR (177.9±155.5 pg/ml; n=9) and MH patients (151.3±96.8 pg/ml; n=9). Double asterisk (**) denotes p<0.01 based on the Kruskal–Wallis test.

We evaluated VEGF concentrations in vitreous body and serum using ELISA (Figure 2). For the statistical analysis, any VEGF level below the limit of detection was set to zero. Intravitreal concentrations of VEGF were much higher (p<0.01) in PDR (798.2±882.7 pg/ml) than in MH (17.7±15.5 pg/ml). Serum VEGF did not differ between the PDR group (177.9±155.5 pg/ml) and the MH group (151.3±96.8 pg/ml; p=0.83).

### Association between EC-SOD and VEGF in vitreous and serum

**Figure 3:**
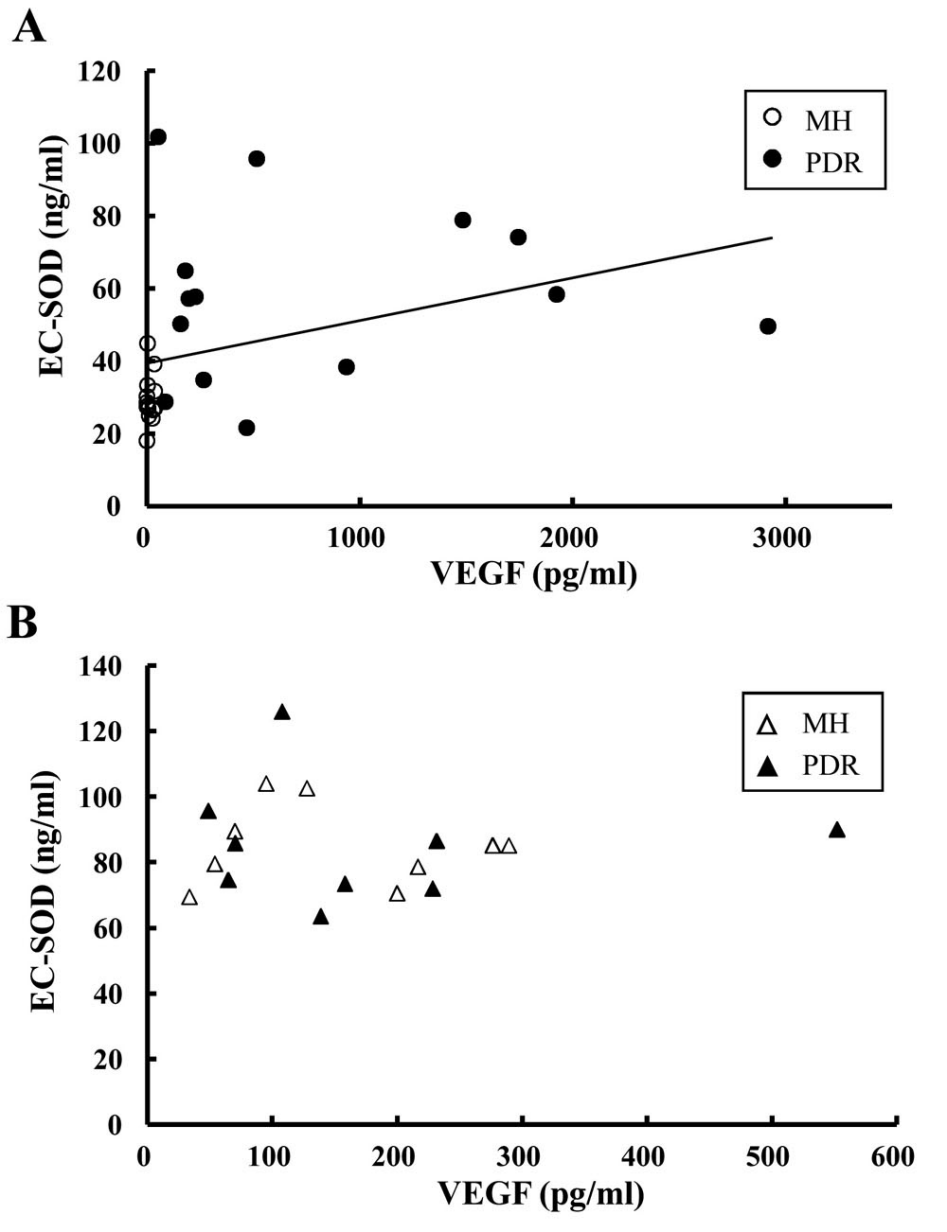
Correlations between EC-SOD and VEGF levels (for vitreous body and serum). **A**: Intravitreal EC-SOD showed a significant correlation with intravitreal VEGF. The correlation coefficient was 0.61, and the p value was p<0.001. **B**: In the serum, there was no significant correlation between EC-SOD and VEGF. For vitreous body data, open circles represent MH patients and closed circles indicate PDR patients. For serum data, open triangles represent MH patients and closed triangles indicate PDR patients. Correlations were examined using Spearman rank-correlation coefficient, and the number of each group was follow: the vitreous samples of PDR (n=14) and MH (n=14), the serum samples of PDR (n=9) and MH (n=9).

We performed association-based analysis on EC-SOD and VEGF levels in vitreous and serum. In all patients (grouped together), intravitreal VEGF showed a significant correlation with intravitreal EC-SOD (rs=0.61, p<0.001; Figure 3A). On the other hand, we did not detect any relation between serum VEGF and serum EC-SOD (rs=-0.03, p=0.46; Figure 3B).

### Effects of EC-SOD on in vitro tube formation in HUVECs

**Figure 4:**
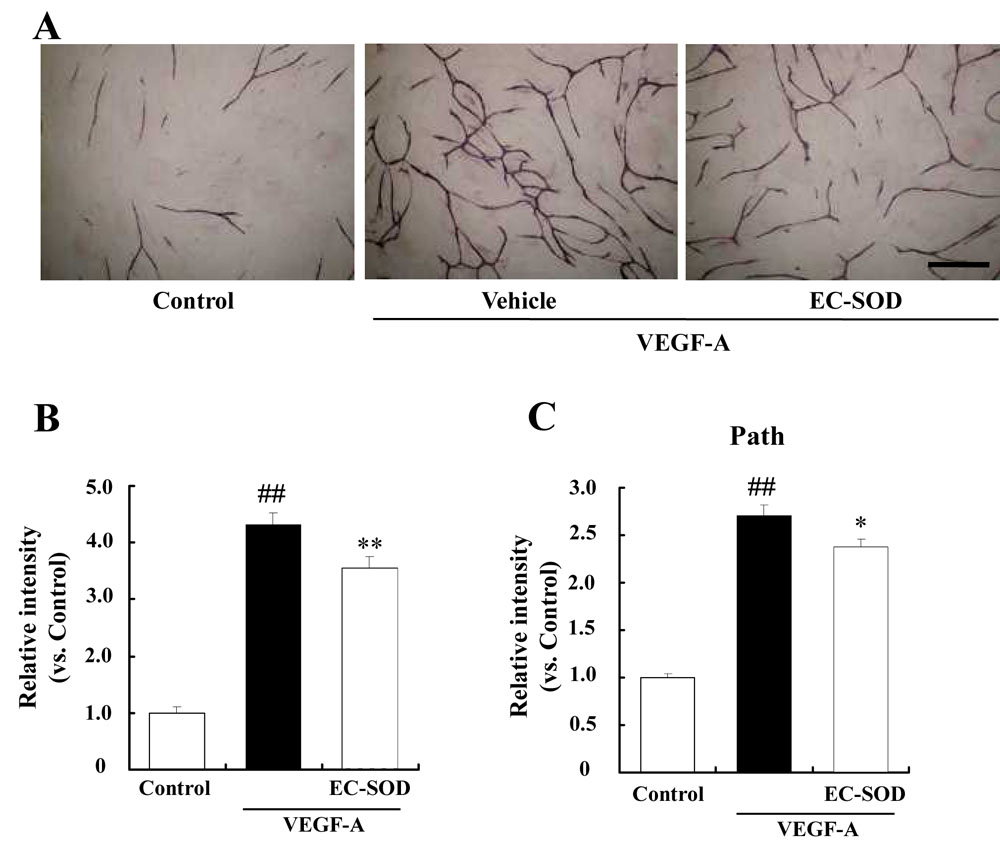
Effects of EC-SOD against in vitro tube formation in HUVECs. **A**: In vitro tube formation was achieved using an in vitro angiogenesis kit. Briefly, HUVECs and fibroblasts were incubated with 10 ng/ml VEGF with or without 100 ng/ml EC-SOD. In the control group, HUVECs were incubated with culture medium. After 11 days, they were stained with anti CD31 antibody, an endothelial cell marker. Scale bar represents 0.5 mm. Tube formation was evaluated by measurements of (**B**) joint and (**C**) path, as described in “Methods.” Data represent means and standard error (n=8), with “Control” being given the value 1.0. Double sharp (##) denotes p<0.01 versus Control based on the Tukey test. Asterisk (*) denotes p<0.05 versus VEGF alone based on the Tukey test. The number of each group was 8.

For insight into the role that might be performed by the upregulated EC-SOD in the vitreous body in PDR patients, we examined the effects of EC-SOD on angiogenesis using an in vitro tube formation model. When treated with VEGF, HUVECs became organized into complex tubular networks, and 100 ng/ml EC-SOD apparently inhibited this effect of VEGF (Figure 4). Two kinds of parameters (joint and path) were increased more than twofold by VEGF treatment (versus control). EC-SOD significantly reduced two parameters of VEGF-induced tube formation. The relative intensities of joint were significantly decre ased in EC-SOD treated group (3.56±0.19) than those of vehicle (4.31±0.21), and the relative intensities of path were significantly decreased in EC-SOD treated group (2.37±0.09) than those of vehicle (2.71±0.11).

### Effects of EC-SOD on VEGF-induced proliferation and migration in HUVECs and HRMVECs

**Figure 5:**
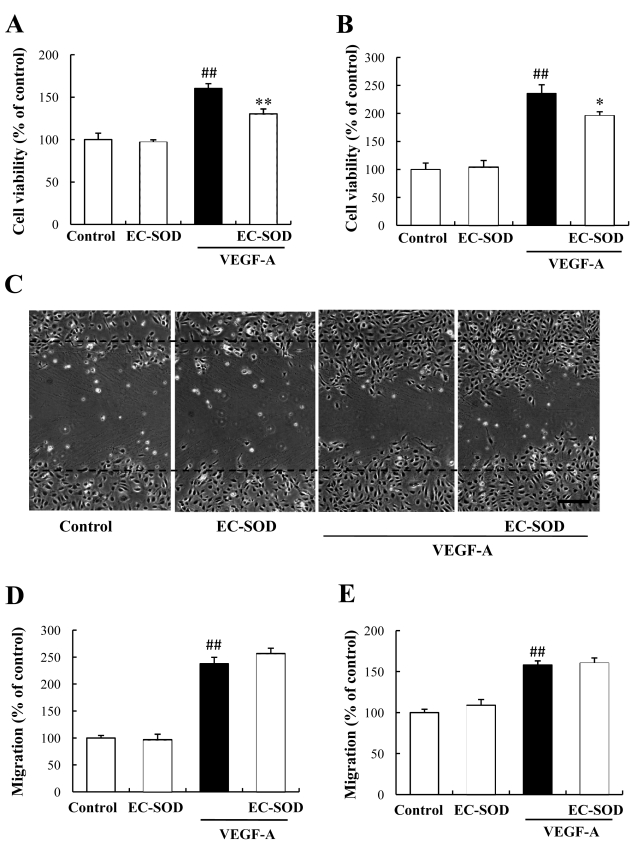
Effects of EC-SOD against VEGF-induced cell proliferation and migration in HUVECs and HRMVECs. In the proliferation assay, (**A**) HUVECs and (**B**) HRMVECs were incubated with 10 ng/ml VEGF with or without 100 ng/ml EC-SOD for 72 h. EC-SOD significantly suppressed VEGF-induced proliferation in HUVECs and HRMVECs. Data represent means and standard error. The number of Control group (n=12), EC-SOD alone (n=12), VEGF alone (n=18), and VEGF plus EC-SOD (n=18). The value of “Control” defines as 100%. Double sharp (##) denotes p<0.01 versus Control based on the Tukey test. Asterisk (*) and double asterisk (**) denotes p<0.05 and p<0.01 versus VEGF alone based on the Tukey test. **C**, **D**: HUVECs and (**E**) HRMVECs migration were assessed using a wound-healing assay. Briefly, 90% confluent monolayer cells were scratch-wounded, then incubated for 24 h. Images of this type of wounded monolayer are shown for 0 h and 24 h after treatment with 10 ng/ml VEGF with or without 100 ng/ml EC-SOD. **C**: These pictures indicated the migration cells after stimulated VEGF with or without EC-SOD in HUVECs. Scale bar represents 250 µm. Horizontal lines indicate wound edges. **D**, **E**: EC-SOD had no effect by itself, and no effect on VEGF-induced migration (versus VEGF alone) in HUVECs and HRMVECs. Data represent means and standard error (n=4). Double sharp (##) denotes p<0.01 versus Control based on the Tukey test.

To investigate the mechanism underlying the above effect of EC-SOD against VEGF-induced tube formation, we examined VEGF-induced cell proliferation and migration in vascular endothelial cells. In the proliferation assay, cell viability in HUVECs and HRMVECs (measured by CCK-8; see Methods) were increased 1.6 fold and 2.1 fold by VEGF (versus control). Despite having no effect by itself, 100 ng/ml EC-SOD significantly suppressed the VEGF-induced cell viability in HUVECs (Figure 5A). Similarly, 100 ng/ml EC-SOD suppressed VEGF-induced cell proliferation in HRMVECs (Figure 5B). Next, we evaluated vascular endothelial cell migration using a wound healing assay. Numbers of HUVECs and HRMVECs increased 1.8 fold and 1.5 fold following VEGF treatment (versus control), but 100 ng/ml EC-SOD did not significantly alter VEGF-induced cell migration (Figure 5D,E).

## Discussion

In the present study, we examined the concentrations of EC-SOD and VEGF in vitreous and serum samples taken from PDR and MH patients. The intravitreal concentrations of EC-SOD and VEGF were significantly higher in PDR patients than in MH patients, and showed a positive correlation with each other (in the whole patient group). However, there were no significant differences between PDR and MH in the serum concentrations of either EC-SOD or VEGF. To investigate possible role performed by the increased intravitreal EC-SOD in PDR, we evaluated the effect of EC-SOD using an in vitro angiogenesis model. We found that EC-SOD significantly suppressed VEGF-induced cell proliferation in HUVECs and HRMVECs, and in vitro tube formation in HUVECs.

In this study, intravitreal EC-SOD and VEGF were significantly higher in PDR patients than in MH patients (Figures 1 and Figure 2). However, the two factors did not correlate (Figure 3). These results suggest that the increase of EC-SOD and VEGF were separately regulated in vitreous body in PDR patients. So, why was EC-SOD increased in the vitreous body in PDR patients? Expression of EC-SOD has been localized to specific cells and tissues, with the highest expression occurring in lung, heart, kidney, and vasculature [15]. Since new vessel formation is promoted in ocular tissue in PDR patients compared with MH patients, upregulation of EC-SOD in ocular tissue is to be expected. However, Coral et al. noted intravitreal levels of homocysteine, a metabolite of methionine, are increased in PDR patients [16]. It has been reported that homocysteine suppresses heparin binding activity of EC-SOD connected with vascular endothelial cells [17]. These results indicate that upregulation of EC-SOD is to be expected in the solution of ocular tissue in PDR patients via increment of newly vessels formation and decrement of heparin binding activity of EC-SOD.

Neovascularization of the retina is a diagnostic feature of PDR and is well correlated with the intravitreal level of VEGF, although the serum VEGF does not change [5,6]. Our data in the present study are in agreement with these previous data [5,6]. The details of the mechanism responsible for the hundredfold elevation of intravitreal VEGF levels in PDR patients compared to nondiabetic controls remain unclear, but it is certain that this type of increase in VEGF in the vitreous body is related to the development of PDR.

A strong relationship exists between diabetic retinopathy and oxidative stress [18]. Verdejo et al. reported that lipid peroxidation markers (malondialdehyde-like metabolites and 4-hydroxynonenal) are increased in the vitreous body of the PDR patient [19]. Oxidative stress is known to be regulated by various redox enzymes (e.g., SOD, catalase, and glutathione-S-transferase) and antioxidants (e.g., vitamin E and coenzyme Q_10_). In the present study, we detected a twofold increase in EC-SOD levels in the vitreous of PDR patients (versus MH). From this result, we hypothesized about the possible role played by the elevated intravitreal level of EC-SOD in PDR, as follows: 1) increased EC-SOD may serve to maintain the equilibrium between oxidative stress and antioxidative activity, a condition in which there is upregulation of lipid oxides in the vitreous body in PDR; or 2) EC-SOD may serve as a inhibitor of angiogenesis. Wheeler et al. noted that overexpression of EC-SOD suppressed implanted B16-F1 tumor cell growth and tumor vascularization in mice [20]. In the present study, EC-SOD suppressed VEGF-induced in vitro angiogenesis by inhibiting cell proliferation (Figures 4 and Figure 5). The effects were confirmed not only in HUVECs but also in HMVECs (Figure 5). It is known that VEGF-induced angiogenesis is mediated by ROS, and various antioxidants suppress the angiogenesis [21]. Taken together, EC-SOD may suppress ROS generation playing the signal pathway, but the detailed mechanism is unclear.

In the present study, external application of EC-SOD at 100 ng/ml partially suppressed VEGF-induced tube formation (both joint and path parameters) in HUVECs (Figure 4), and proliferation in HUVECs and HMVECs (Figure 5). EC-SOD at 100 ng/ml exhibits angiostatic effect in an in vitro experiment, the concentration of which consistents with the intravitreal concentration in PDR patients (20 to 100 ng/ml). Hence, the angiostatic effect of EC-SOD was weak, but the effect may reflect the acute intravitreal environment of PDR patients.

The loss of EC-SOD from the vasculature of diabetic patients may have implications with regard to the interaction of superoxide and nitric oxide (NO) in blood vessels. Although glycation of EC-SOD does not affect enzymatic activity [22], glycation does inhibit the activity of the CuZn-SOD [23]. Heparin affinity of EC-SOD can be reduced in diabetic conditions, because of high blood glucose, through nonenzymatic glycation of EC-SOD at lysine residues located in the heparin-binding domain [22]. Our present study revealed that intravitreal levels of EC-SOD were increased in PDR compared with nondiabetic MH patients. This increase may reflect the alteration of EC-SOD from the extracellular matrix to the interstitium in the diabetic condition.

The mediating roles that oxidants play between VEGF overexpression and diabetic retinopathy are well established. Substances that decrease ROS levels may well prove to be useful in treating diabetic retinopathy. For example, low molecular weight SOD and catalase mimetics are highly effective at catalyzing the transformation of superoxide and hydrogen peroxide [24]; however, tempol, a SOD mimetic, can reverse endothelial dysfunction in diabetic rats [25], and FP15, a peroxynitrite decomposition catalyst, has been reported to protect against both leukocyte adhesion within retinal vessels and vascular dysfunction in diabetic mice [26]. As ocular tissues may be heavily exposed to free radicals in diabetic and nondiabetic conditions, treatment aimed at increasing antioxidative activity may have beneficial effects against diabetic retinopathy.

NO promotes cell migration in endothelial cells. NO reacts at an almost diffusion-controlled rate with superoxide resulting in loss of NO bioactivity [27]. Hence, EC-SOD may be important role for maintaining the NO bioactivity. In the present study, EC-SOD suppressed VEGF-induced cell proliferation and tube formation, but not migration, in HUVECs (Figures 4 and Figure 5). At the present time it is unclear why EC-SOD did not affect VEGF-induced migration in HUVECs and HRMVECs, and thus further investigations are warranted.

In the present study, we measured the intravitreal concentrations of VEGF in PDR patients. Some patients with PDR also experienced vitreous hemorrhage. To investigate the effects of vitreous hemorrhage, we compared intravitreal levels of VEGF from PDR patients with or without the hemorrhage condition. However, we found no significant differences between hemorrhage and nonhemorrhage groups. Similarly, intravitreal levels of EC-SOD did not differ significantly between the hemorrhage and non-hemorrhage groups (data not shown). Hence, intravitreal levels of VEGF and EC-SOD were not influenced by vitreous hemorrhage in PDR patients.

In conclusion, our clinical study revealed that EC-SOD and VEGF were increased in the vitreous body from PDR patients. EC-SOD may be related to upregulation of VEGF levels in the vitreous body during PDR, suggesting that EC-SOD may play a pivotal role in the pathogenesis of angiogenesis.

## References

[1] Senger DR Galli SJ Dvorak AM Perruzzi CA Harvey VS Dvorak HF Tumor cells secrete a vascular permeability factor that promotes accumulation of ascites fluid. Science 1983 219 983 5 6823562 10.1126/science.6823562

[2] Keck PJ Hauser SD Krivi G Sanzo K Warren T Feder J Connolly DT Vascular permeability factor, an endothelial cell mitogen related to PDGF. Science 1989 246 1309 12 2479987 10.1126/science.2479987

[3] Millauer B Shawver LK Plate KH Risau W Ullrich A Glioblastoma growth inhibited in vivo by a dominant-negative Flk-1 mutant. Nature 1994 367 576 9 8107827 10.1038/367576a0

[4] Sone H Kawakami Y Okuda Y Kondo S Hanatani M Suzuki H Yamashita K Vascular endothelial growth factor is induced by long-term high glucose concentration and up-regulated by acute glucose deprivation in cultured bovine retinal pigmented epithelial cells. Biochem Biophys Res Commun 1996 221 193 8 8660335 10.1006/bbrc.1996.0568

[5] Aiello LP Clinical implications of vascular growth factors in proliferative retinopathies. Curr Opin Ophthalmol 1997 8 19 31 10168890 10.1097/00055735-199706000-00005

[6] Funatsu H Yamashita H Mimura T Noma H Nakamura S Hori S Risk evaluation of outcome of vitreous surgery based on vitreous levels of cytokines. Eye 2007 21 377 82 16410812 10.1038/sj.eye.6702213

[7] Sies H Oxidative stress: oxidants and antioxidants. Exp Physiol 1997 82 291 5 9129943 10.1113/expphysiol.1997.sp004024

[8] Brownlee M Biochemistry and molecular cell biology of diabetic complications. Nature 2001 414 813 20 11742414 10.1038/414813a

[9] Ellis EA Grant MB Murray FT Wachowski MB Guberski DL Kubilis PS Lutty GA Increased NADH oxidase activity in the retina of the BBZ/Wor diabetic rat. Free Radic Biol Med 1998 24 111 20 9436620 10.1016/s0891-5849(97)00202-5

[10] Obrosova IG Minchenko AG Marinescu V Fathallah L Kennedy A Stockert CM Frank RN Stevens MJ Antioxidants attenuate early up regulation of retinal vascular endothelial growth factor in streptozotocin-diabetic rats. Diabetologia 2001 44 1102 10 11596663 10.1007/s001250100631

[11] Ushio-Fukai M Tang Y Fukai T Dikalov SI Ma Y Fujimoto M Quinn MT Pagano PJ Johnson C Alexander RW Novel role of gp91(phox)-containing NAD(P)H oxidase in vascular endothelial growth factor-induced signaling and angiogenesis. Circ Res 2002 91 1160 7 12480817 10.1161/01.res.0000046227.65158.f8

[12] el-Remessy AB Bartoli M Platt DH Fulton D Caldwell RB Oxidative stress inactivates VEGF survival signaling in retinal endothelial cells via PI 3-kinase tyrosine nitration. J Cell Sci 2005 118 243 52 15615788 10.1242/jcs.01612

[13] Fattman CL Schaefer LM Oury TD Extracellular superoxide dismutase in biology and medicine. Free Radic Biol Med 2003 35 236 56 12885586 10.1016/s0891-5849(03)00275-2

[14] Adachi T Nakamura M Yamada H Futenma A kato K, Hirano K. Quantitative and qualitative changes of extracellular-superoxide dismutase in patients with various diseases. Clin Chim Acta 1994 229 123 31 7988042 10.1016/0009-8981(94)90234-8

[15] Zelko IN Mueller MR Folz RJ Transcription factors sp1 and sp3 regulate expression of human extracellular superoxide dismutase in lung fibroblasts. Am J Respir Cell Mol Biol 2008 39 243 51 18314536 10.1165/rcmb.2007-0378OC PMC2542458

[16] Coral K Angayarkanni N Gomathy N Bharathselvi M Pukhraj R Rupak R Homocysteine levels in the vitreous of proliferative diabetic retinopathy and rhegmatogenous retinal detachment: its modulating role on lysyl oxidase. Invest Ophthalmol Vis Sci 2009 50 3607 12 19369240 10.1167/iovs.08-2667

[17] Yamamoto M Hara H Adachi T Effects of homocysteine on the binding of extracellular-superoxide dismutase to the endothelial cell surface. FEBS Lett 2000 486 159 62 11113458 10.1016/s0014-5793(00)02260-2

[18] Caldwell RB Bartoli M Behzadian MA El-Remessy AE Al-Shabrawey M Platt DH Liou GI Caldwell RW Vascular endothelial growth factor and diabetic retinopathy: role of oxidative stress. Curr Drug Targets 2005 6 511 24 16026270 10.2174/1389450054021981

[19] Verdejo C Marco P Renau-Piqueras J Pinazo-Duran MD Lipid peroxidation in proliferative vitreoretinopathies. Eye 1999 13 183 8 10450379 10.1038/eye.1999.48

[20] Wheeler MD Smutney OM Samulski RJ Secretion of extracellular superoxide dismutase from muscle transduced with recombinant adenovirus inhibits the growth of B16 melanomas in mice. Mol Cancer Res 2003 1 871 81 14573788

[21] Colavitti R Pani G Bedogni B Anzevino R Borrello S Waltenberger J Galeotti T Reactive oxygen species as downstream mediators of angiogenic signaling by vascular endothelial growth factor receptor-2/KDR. J Biol Chem 2002 277 3101 8 11719508 10.1074/jbc.M107711200

[22] Adachi T Ohta H Hayashi K Hirano K Marklund SL The site of nonenzymic glycation of human extracellular-superoxide dismutase in vitro. Free Radic Biol Med 1992 13 205 10 1505778 10.1016/0891-5849(92)90016-a

[23] Kawamura N Ookawara T Suzuki K Konishi K Mino M Taniguchi N Increased glycated Cu,Zn-superoxide dismutase levels in erythrocytes of patients with insulin-dependent diabetis mellitus. J Clin Endocrinol Metab 1992 74 1352 4 1592880 10.1210/jcem.74.6.1592880

[24] Hoogwerf BJ Young JB The HOPE study. Ramipril lowered cardiovascular risk, but vitamin E did not. Cleve Clin J Med 2000 67 287 93 10780101 10.3949/ccjm.67.4.287

[25] Nassar T Kadery B Lotan C Da'as N Kleinman Y Haj-Yehia A Effects of the superoxide dismutase-mimetic compound tempol on endothelial dysfunction in streptozotocin-induced diabetic rats. Eur J Pharmacol 2002 436 111 8 11834254 10.1016/s0014-2999(01)01566-7

[26] Sugawara R Hikichi T Kitaya N Mori F Nagaoka T Yoshida A Szabo C Peroxynitrite decomposition catalyst, FP15, and poly(ADP-ribose) polymerase inhibitor, PJ34, inhibit leukocyte entrapment in the retinal microcirculation of diabetic rats. Curr Eye Res 2004 29 11 6 15370362 10.1080/02713680490513146

[27] Thomson L Trujillo M Telleri R Radi R Kinetics of cytochrome c2+ oxidation by peroxynitrite: implications for superoxide measurements in nitric oxide-producing biological systems. Arch Biochem Biophys 1995 319 491 7 7786032 10.1006/abbi.1995.1321

